# Macrophages in age-related chronic inflammatory diseases

**DOI:** 10.1038/npjamd.2016.18

**Published:** 2016-07-28

**Authors:** Yumiko Oishi, Ichiro Manabe

**Affiliations:** 1 Department of Cellular and Molecular Medicine, Medical Research Institute, Tokyo Medical and Dental University, Tokyo, Japan; 2 Department of Aging Research, Graduate School of Medicine, Chiba University, Chiba, Japan

## Abstract

Chronic inflammation is the common pathological basis for such age-associated diseases as cardiovascular disease, diabetes, cancer and Alzheimer’s disease. A multitude of bodily changes occur with aging that contribute to the initiation and development of inflammation. In particular, the immune system of elderly individuals often exhibits diminished efficiency and fidelity, termed immunosenescence. But, although immune responses to new pathogens and vaccines are impaired, immunosenescence is also characterized by a basal systemic inflammatory state. This alteration in immune system function likely promotes chronic inflammation. Changes in the tissue microenvironment, such as the accumulation of cell debris, and systemic changes in metabolic and hormonal signals, also likely contribute to the development of chronic inflammation. Monocyte/macrophage lineage cells are crucial to these age-associated changes, which culminate in the development of chronic inflammatory diseases. In this review, we will summarize the diverse physiological and pathological roles of macrophages in the chronic inflammation underlying age-associated diseases.

## Introduction

With advancing age, the immune system undergoes a dynamic change characterized by the coexistence of a smaller immune response to newly encountered pathogens or vaccine antigens, and an elevated systemic inflammatory state made manifest, for example, by elevated levels of proinflammatory cytokines, clotting factors and acute phase reactants.^1^ This chronic activation of inflammation associated with aging has been termed inflammaging, and recent studies indicate it is involved in the development of such non-communicable diseases (NCDs) as cardiovascular and metabolic disease and cancer in the elderly. Although the chronic inflammation associated with NCDs does not necessarily lead by age-associated changes in the body, the observation that the prevalence of many NCDs increases with advancing age suggests a pathogenic link between inflammaging and age-associated diseases.

Chronic inflammation is a prolonged condition in which tissue injury and attempts at repair coexist, leading to tissue remodeling and dysfunction.^2^ Although chronic inflammation may follow acute inflammation, in the most common NCDs of today it likely begins insidiously as a low-grade, smoldering response with no manifestation of the cardinal signs of inflammation (Dolor (pain), Calor (heat), Rubor (redness) and Tumor (swelling)). However, even low-grade inflammation may impair tissue function (Functio laesa). For instance, inflammatory signals interfere with insulin signaling.^3^ Moreover, the continuous progression of tissue injury and repair promotes tissue remodeling (e.g., extensive fibrosis) that may eventually cause irreversible tissue dysfunction.^4^ In fact, the severity of tissue remodeling determines the prognosis of some NCDs, such as heart failure and chronic kidney disease.^5^

A complex interplay between parenchymal cells within a tissue and the various cells in the stroma, including immune cells, vascular cells and fibroblasts, lead the processes of chronic inflammation under the influence of inputs from both the local microenvironment and the wider system. Of particular interest are monocyte–macrophage lineage cells, which act as major effector cells in chronic inflammatory processes during the pathological development of NCDs.^4^ In this review, we will summarize the pathological connection between chronic inflammation and age-associated diseases, with a particular focus on what is currently known about the roles played by macrophages.

## Immunosenescence and age-associated diseases

It is often noted that elderly individuals are more vulnerable to infectious diseases. For instance, occult infection with tuberculosis and varicella zoster virus becomes evident, sometimes leading to life-threatening disease. Moreover, vaccines are often ineffective in older adults owing to inability of the adaptive immune system to generate protective immunity. Overall, these changes in the immune system, characterized by declining fidelity and efficiency, are termed immunosenescence. A key feature of immunosenescence is an imbalance between inflammatory and anti-inflammatory networks, leading to a complex presentation of impaired adaptive immune responses with concomitant persistent low-grade inflammation and a greater susceptibility to autoimmune responses.^6^ The changes in the adaptive immune system are characterized by a decreases in naive T- and B cells, an increase in memory cells and a progressive reduction in the T-cell receptor (TCR) and B-cell receptor (BCR) repertoire.^7–9^ By contrast, generation of myeloid cells is favored in aging animals.

Immunosenescence involves not only age-related changes intrinsic to immune cells, but also microenvironmental and systemic alterations. Although it is likely that cellular senescence is involved in some of these age-related alterations, many are likely induced independently of cellular senescence pathways. In addition, cellular senescence in non-immune cells may also contribute to immunosenescence.^10^ Thus, complex mechanisms at multiple levels (e.g., cellular, tissue and systemic levels) appear to contribute to the development of immunosenescence.

Multiple causes have been suggested for the age-related functional impairment of lymphocytes, including qualitative changes in hematopoietic stem cells (HSCs) and progenitor cells, resulting in reduced production of T- and B lymphocytes in bone marrow and the thymus,^11^ as well as changes in non-lymphocyte compartments of the thymus. Elderly HSCs exhibit less potential to produce lymphocytes and an increase in myeloid potential.^6^ Distinct HSC subsets having lymphoid- or myeloid-biased potential have been identified,^12^ and while the number of lymphoid-biased HSCs declines with age, myeloid-biased HSCs are increased. This shift toward myelopoiesis is thought to reflect the influence of both intrinsic and extrinsic factors.^13^ For instance, transforming growth factor (TGF)-β1 promotes proliferation of myeloid-biased HSCs and myeloid progenitors,^12^ suggesting TGF-β1 may be one of the bone marrow microenvironmental factors altered with age. Decreased production of interleukin (IL)-7 by stromal cells may also contribute to the age-related reduction in B-cell progenitors.^14^ Age-related microenvironmental changes also impair T-cell development in the thymus. Indeed, age-associated thymal regression resulting in reduced production of naive T cells is now thought to be driven by the epithelial compartment.^10^ In addition to the local environment, systemic changes that occur with aging, such as decreased levels of growth hormone and IGF-1, may also contribute to the decline in lymphopoiesis.^11^ Despite the skewing of aged HSCs toward myelopoiesis, however, the basal inflammation seen in older individuals is not associated with increased numbers of myeloid cells, presumably reflecting multiple functional defects in the homing and proliferative responses of aged HSCs.^1,15^

Immunosenescence leads not only to impaired immune responses but also to low-grade, chronic, systemic inflammation (inflammaging).^16^ And this chronic inflammatory activation, which has been noted in tissues from aged humans and mice,^17–19^ may have a role in the development of a plethora of age-associated chronic diseases, including cardiovascular and metabolic diseases and cancer. Other factors, including changes in the microenvironment as well as systemic factors, also contribute to chronic inflammation. For instance, senescent cells in tissues may activate inflammatory responses by recruiting immune cells by expressing senescence-associated secretory phenotypes (SASPs).^20^ Increased cell death or ineffective clearance of dead cells and damaged tissue may also activate immune responses.^21^

With chronic inflammation, innate immune cells act as key effector cells. In the following sections, we will focus on age-related changes in the innate immune system, particularly macrophages, to address how aging paradoxically promotes chronic inflammation.

## Innate immune cells in the control of inflammaging

**Figure 1:**
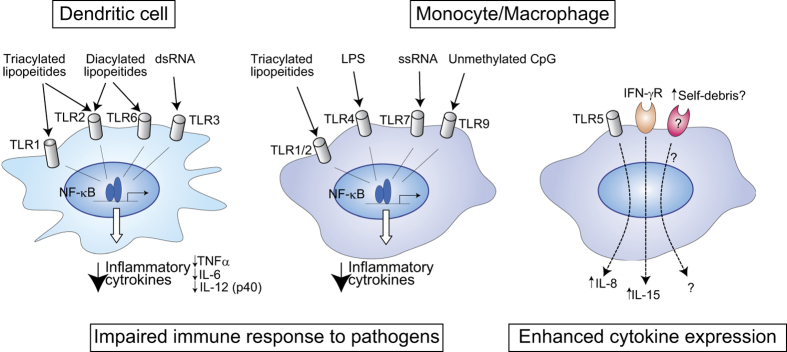
Aberrant inflammatory response of a dendritic cell and macrophage with aging. Toll-like receptors (TLRs) are a family of pattern-recognition receptors that have a key role in the innate immune system. TLRs are activated by specific ligands derived from pathogens and damaged cells, as shown. The expression and function of some TLRs are downregulated with aging, potentially impairing immune responses. On the contrary, signaling mediated via TLR and IFNγ is activated, resulting in increased secretion of inflammatory cytokines. IFN, interferon; LPS, lipopolysaccharide; dsRNA, double-stranded RNA; ssRNA, single-stranded RNA.

The innate immune system serves as the body’s immediate first line of defense, which studies in both animal models and humans have shown is altered with aging. In addition to the developmental shift toward the myeloid lineage of HSCs,^15^ age-related dysfunction of neutrophils, natural killer cells, monocytes, macrophages and dendritic cells has been reported.^22–25^ For instance, the capacity to activate T cells via antigen presentation is impaired in aged dendritic cells, where expression of costimulatory molecules and MHC class II is reportedly downregulated, though the conflicting data have also been reported.^26^ Dendritic cells from elderly subjects also express lower levels of Toll-like receptors (TLRs) and produce lower levels of cytokines in response to specific TLR ligands, while the baseline cytokine levels in absence of TLR ligand stimulation are increased, suggesting dysregulated cytokine expression.^26,27^ Similarly, in blood monocytes from aged human subjects, TLR1/2-induced tumor necrosis factor (TNF)-α and IL-6 production is decreased, suggesting a functional defect in monocyte/macrophage lineage cells (Figure 1).^28^ In addition, phagocytosis by monocytes is impaired in older individuals,^25^ and aged macrophages exhibit reduced chemotaxis, reduced expression of MHC class II and less capacity for antigen presentation.^29^ Neutrophils also become dysfunctional in aged individuals.^30^ This age-associated dysfunction of innate immune cells likely contributes to impaired immune responses to vaccines and infections, as well as to the increases in morbidity and mortality noted in elderly populations.^31^

On the other hand, not every inflammatory response is diminished with aging. For instance, human monocytes from older individuals express higher levels of intracellular TNF-α at baseline and after lipopolysaccharide treatment.^25^ TLR5 expression, ligand-induced IL-8 production and IFN-γ-mediated IL-15 production are all elevated in monocytes from elderly subjects (Figure 1).^24,32^ These altered, aberrant signals may in part provide the mechanism underlying the elevated serum inflammatory cytokine levels and greater propensity for chronic inflammation that comes with aging.

In addition to the intrinsic dysregulation of innate immune cells, the altered microenvironment within aged tissues and systemic changes in the endocrine and other systems very likely contribute to aberrant activation of innate immune cells. The innate immune system recognizes the repetitive molecular structures of pathogens, known as pathogen-associated molecular patterns (PAMPs), via pattern-recognition receptors.^33^ Cells involved in the innate immune system (e.g., macrophages) express a variety of pattern-recognition receptors, including TLRs. Upon recognition of a PAMP, innate immune cells are activated to destroy the pathogen and/or pathogen-infected cells. Although PAMPs are exogenous molecules, the damaged or dying cells release endogenous molecules called damage/danger-associated molecular patterns (DAMPs), which also activate the immune system in a manner analogous to PAMPs. A variety of different molecules have now been identified as DAMPS, including high-mobility group box 1 protein (HMGB1), genomic double-stranded DNA and cleaved extracellular matrix proteins.^34^ Moreover, modified endogenous molecules such as oxidized lipoproteins, cholesterol crystals and uric acid, whose production does not necessarily require cell death, may also serve as DAMPs.^35,36^ Those and still other DAMPs, such as damaged macromolecules and cell debris, accumulate with age, leading to activation of the innate immune system. This accumulation of DAMPs may be enhanced by the inadequate phagocytotic activity of aged myeloid cells.^1^ Thus, a complex reciprocal interplay between immune cells and tissue microenvironmental/non-immune cells very likely contributes to the activation of inflammatory signaling in aged individuals.

Systemic factors, such as hormonal and metabolic changes, also impact immune responses. For instance, menopause and ovariectomy cause a low-grade systemic inflammatory state with increased plasma chemokine levels that is suppressed by estrogen treatment.^37^ Estrogen can activate or inhibit proinflammatory cytokine production, depending on context and dose.^38^ Obesity associates with activation of chronic low-level inflammation in several tissues, particularly adipose tissue.^39^ Conversely, calorie restriction without malnutrition suppresses chronic inflammation.^40^ Malnutrition, on the other hand, leads to immune dysfunction.^41^ Hormones that control metabolism, such as insulin and leptin, are known to modulate immune cell function.^42^ Obesity accelerates immunosenescence in T cells,^43^ and serum cytokines levels are increased in obese subjects.^44,45^ Aging associates with an increase in body fat and a shift of fat from subcutaneous to abdominal depots.^46^ The related systemic metabolic changes likely contribute to the basal activation of inflammation in the elderly. We will discuss later how obesity induces inflammation within visceral adipose tissue and how adipose tissue inflammation not only impacts systemic metabolism but also promotes chronic inflammatory disease.

## Chronic inflammation: failed resolution of inflammation

Acute inflammation is a protective response to injury or infection.^4^ Acute inflammation is typically self-limiting, and after the offending agent is eliminated the tissue returns to the homeostatic state. The resolution of acute inflammation is an active process in which anti-inflammatory signals suppress inflammation, clear immune cells and promote healing, leading to the restoration of normal tissue function.^47^ Because the resolution involves dynamic processes, dysfunctional resolution prolongs and perpetuates inflammation. Many mechanisms can cause impaired resolution and sustained inflammation—failure to eliminate the offending agent, for instance. Insufficient clearance of proinflammatory cells may also hamper resolution, and functional alteration of immune cells may affect resolution-related signaling and processes. It can be inferred from this that in aged tissue, accumulation of DAMPs due to their increased production and/or impaired clearance may predispose one to chronic inflammation.

A primary function of macrophages during inflammatory resolution is the clearance of effete cells. Particularly important is their phagocytosis of apoptotic granulocytes.^48^ In a mouse self-resolving peritonitis model, resolution of acute inflammation was delayed in aged mice.^49^ Numbers of granulocytes and levels of proinflammatory cytokines were higher in aged mice than young mice 24 h after peritonitis was triggered. Moreover, although the numbers of monocytes/macrophages in peritoneal exudates were increased in aged mice, bone marrow-derived macrophages from aged mice showed less capacity to take up apoptotic granulocytes, suggesting diminished macrophage activity may contribute to failed resolution of inflammation. Impaired phagocytosis of apoptotic cells has also been noted in other *in vivo* mouse models and in human monocyte-derived dendritic cells *in vitro.*^50,51^ One study also suggests that aging impairs the phagocytotic capacity of microglia.^52^ In addition, macrophages from obese ob/ob mice exhibit a diminished ability to phagocytose apoptotic cells,^53^ suggesting that systemic metabolic dysfunction, which is often seen in the elderly, may further impair phagocytotic capacity. This may mean that diminished macrophage phagocytotic activity results in failed resolution and accumulation of DAMPs in aged animals. However, that notion needs to be directly tested in future studies. It will also be important to determine mechanistically how aging causes loss of phagocytotic functionality. A previous observation that apoptotic clearance by thioglycollate-elicited peritoneal macrophages from young mice was impaired after the cells were exposed to serum from aged mice suggests exogenous factors contribute to the age-related decline in phagocytotic activity.^50^ On the other hand, the finding that dendritic cells generated *in vitro* from human monocytes exhibited age-associated phagocytotic dysfunction with impairment of the PI3K/AKT pathway^51^ suggests a cell-autonomous mechanism. Presumably, both intrinsic and extrinsic mechanisms drive the functional alterations in macrophages and reflect the cells’ highly plastic gene expression, as we will discuss in the following sections.

## Cellular senescence, clearance and macrophages

Macrophages are also essential for clearing senescent cells.^54^ It has been suggested that accumulation of senescent cells within tissues contributes to age-related organ dysfunction and pathology. Baker *et al.*^51^ showed that removing cells expressing the senescence-marker gene *Cdkn2a* (p16) in a progeroid context (*BubR1*^H/H^ mice) delays the onset of age-related phenotypes, including sarcopenia, cataracts and loss of adipose tissue, which highlights the importance of proper clearance of senescent cells. Very recently, the same group showed that partial ablation of p16^+^ cells extended lifespan and healthspan in a non-progenoid context as well, and this was accompanied by reduced expression of inflammation markers in various tissues.^56^

**Figure 2:**
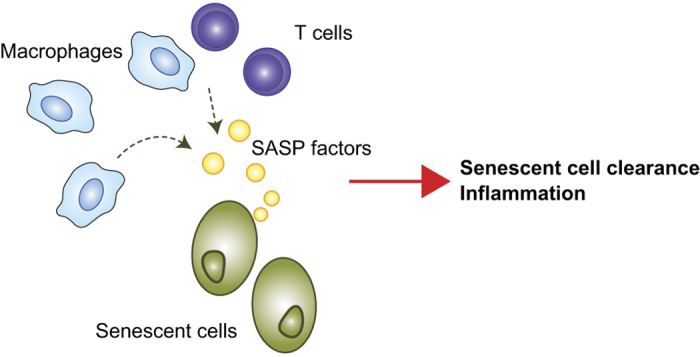
Role of macrophages in the clearance of senescent cells. Senescent cells secrete proinflammatory mediators, senescence-associated secretory phenotype (SASP) factors. SASP factors recruit immune cells (macrophages, neutrophils and T cells), which then clear the senescent cells. In addition, CD4^+^ T cells are known to survey the antigens expressed in premalignant senescent hepatocytes and interact with monocytes/macrophages to clear those senescent cells.^115^ Note that the biological effects of SASP factors are not limited to the recruitment of immune cells. SASP factors may promote cell proliferation, differentiation and migration as well as extracellular matrix remodeling. Consequently, SASP factors likely contribute to tissue regeneration and healing. However, they may also promote inflammation in age-associated pathologies.

Senescent cells produce proinflammatory mediators and proteases in what is collectively termed the SASP.^57^ SASP factors recruit immune cells, including macrophages, neutrophils, natural killer cells and T cells, and promote inflammation (Figure 2). This inflammatory response appears to be crucial for clearance of senescent cells by immune cells and for preparing the environment for regeneration and tissue renewal. In that sense, SASP-induced recruitment of immune cells is an essential physiological process that eliminates unwanted cells and leads to developmentally and physiologically necessary tissue remodeling.^20^ However, if these proinflammatory signals and inflammatory processes are not properly regulated, they may promote pathological inflammation. Recent studies suggest SASPs promote pathology in age-associated diseases. For instance, senescent smooth muscle cells express SASP factors in the form of cytokines and chemokines that recruit macrophages and induce proinflammatory phenotypes in endothelial cells *in vitro*. This suggests senescent smooth muscle cells promote vascular inflammation.^58^ Within adipose tissue, senescent preadipocytes accumulate with aging in both humans and rats.^59^ Radiation-induced senescence in preadipocytes activates production of a number of proinflammatory cytokines *in vitro*, while JAK inhibition suppresses *in vitro* expression of SASP factors and alleviates adipose tissue and systemic inflammation in aged mice. This suggests JAK-mediated production of SASP factors contributes to adipose tissue inflammation. However, the extent to which inhibition of SASPs expressed in senescent adipocytes contributes to the observed changes remains unclear, as JAK inhibitors also likely interfere with pathways unrelated to cellular senescence. Because the inflammatory mediators (SASP factors) produced by senescent cells are involved in chronic inflammation and tissue dysfunction (e.g., insulin resistance) in age-associated diseases, it has been suggested that cellular senescence contributes to the development of these diseases by inducing SASP expression. Further studies will be needed to determine the extent to which SASPs contribute to the initiation and development of chronic inflammation in the elderly and to clarify the causal link between cellular senescence, SASPs and age-related pathologies.

It would also be expected that insufficient clearance of senescent cells would prolong inflammatory processes owing to the production of SASP factors and accumulation of DAMPs. Other types of cell death may also contribute to the prolongation of inflammatory processes. For instance, improper clearance of apoptotic cells may contribute to the pathogenesis of age-associated diseases as well as autoimmune diseases. Apoptotic cells are normally rapidly phagocytosed, preventing the leakage of their immunogenic and cytotoxic intracellular contents.^60^ If, however, apoptotic cells are not promptly cleared, they undergo secondary necrosis, leading to the leakage of molecules that elicit inflammatory responses by acting as DAMPs and autoantigens.

Atherosclerosis is a chronic inflammatory disease in which unresolved inflammation promotes vessel wall remodeling and destabilization of atherosclerotic plaque. Improper clearance of apoptotic cells by macrophages (efferocytosis) is one of the mechanisms thought to underlie the unresolved inflammation in atherosclerosis. Within atherosclerotic plaque, macrophages take up modified lipoproteins and become foam cells. As the plaque progresses, foam cell apoptosis overwhelms the remaining macrophages’ capacity for phagocytotic clearance.^61^ Secondary necrosis of the foam cells leads to the expansion of a necrotic core that consists primarily of debris from dead macrophages.^62^ This necrotic debris also promotes inflammation and destabilization of the plaque, which can lead to plaque rupture.^63^ It is currently unclear whether macrophage apoptosis is accelerated by aging, but peritoneal macrophages from aged mice are more susceptible to endoplasmic reticulum stress-induced apoptosis.^64^ The age-related changes in macrophage function may thus contribute to the progression of atherosclerosis. Cellular senescence of endothelial cells and smooth muscle cells also likely contribute to the inability to resolve inflammation in atherosclerosis.^65^

## Macrophage diversity in chronic inflammation

Monocyte–macrophage lineage cells are multifunctional and found in nearly all tissues throughout the body. In addition to their essential roles in host defense, they are pivotally involved in the maintenance of tissue homeostasis. As we have discussed, macrophages are essential for proper remodeling and healing after tissue injury. Phagocytosis by macrophages is also indispensable for tissue remodeling during organ development and ontogeny.

Macrophages are often divided into two subgroups: M1 and M2.^66^ Although this dichotomy is widely used, it is important to keep in mind that the M1/M2 dichotomy is clearly not sufficient to encompass the diverse phenotypes and functions of macrophages, and that classification of macrophages into these two groups is often not straightforward and relies on different sets of markers in different tissues. Exposure to TLR ligand or Th1 cytokines, such as TNF-α and IFN-γ, polarizes macrophages into the proinflammatory, M1 phenotype. M1 activation increases expression of proinflammatory cytokines and production of reactive oxygen species. By contrast, Th2 cytokines, such as IL-4 and IL-13, induce the M2 phenotype, though other factors may be involved.^67^ M2 macrophages are known to be essential for parasite clearance and have also been shown to promote resolution of inflammation and fibrosis.

The different functions of M1 and M2 macrophages have been demonstrated in various tissues relevant to NCDs. For instance, following renal injury the initially recruited monocytes preferentially differentiate into M1 macrophages and promote inflammation.^68,69^ Later, however, they preferentially assume the M2 phenotype and promote fibrosis. Similar transitions of macrophage phenotypes occur after skeletal muscle injury or myocardial infarction.^70,71^ In these models M2 macrophages are increased through recruitment of monocytes and/or *in situ* proliferation of resident macrophages.^68,72^

Aging may modulate M1/M2 activation and polarization.^73,74^ SASP factors secreted from hepatic stellate cells shift macrophage polarization from M2 toward M1.^75^ Accordingly, it is likely that exogenous changes in the microenvironment (e.g., SASP factor secretion) in combination with the systemic low-grade, inflammatory state modulate macrophage activation programs and their responses to stimuli.

Monocyte–macrophage lineage cells constitutively localized in peripheral tissues are called tissue-resident macrophages and exhibit highly diverse and plastic phenotypes that depend on the tissue type. Recent studies have shown that in addition to monocyte-derived macrophages, tissue-resident macrophages crucially contribute to age-related pathology. For instance, a recent study found that an age-dependent increase in reactive oxygen species in mitochondria and the associated NLRP3 inflammasome activation contributed to the pathogenesis of fibrosis in aged mice.^76^

As we will discuss in the following section, the origins of tissue-resident macrophages may differ among tissues and may be affected by aging. As such, different origins add another level of complexity to macrophage diversity.

## Macrophage lineage cells in metabolic control

Macrophages variously contribute to the physiology and pathology of metabolic tissues (Table 1). For example, Küpffer cells, resident macrophages in the liver, activate fatty acid β oxidation in hepatocytes and maintain metabolic homeostasis in the liver.^77^ On the other hand, inflammatory activated Kupffer cells secrete proinflammatory cytokines, likely promoting insulin resistance.^78,79^ High-fat diet-induced obesity induces chronic, low-grade inflammation in the hypothalamus accompanied by activation of microglia, the resident macrophages in the central nervous system.^80^ This hypothalamic inflammation increases appetite by modulating the balance between anorexic and orexigenic neuron activities. Within pancreatic islets, resident macrophages are important for β-cell development, and M2-type islet macrophages have been shown to have a protective role in pancreatitis models.^81^ On the other hand M1 macrophages promote β-cell dysfunction.^82^

Macrophages also have diverse and pivotal roles within adipose tissue. Visceral obesity has been shown to promote both cardiovascular and metabolic disease. Inflammation within visceral adipose tissue both modulates the function of adipose tissue locally and promotes systemic metabolic dysfunction. For example, when secreted by immune cells within adipose tissue, inflammatory cytokines such as TNF-α not only alter local adipocyte function but they also act systemically to induce insulin resistance. In addition, adipose tissue-derived proinflammatory cytokines may promote atherosclerosis and cancer by activating inflammatory processes in responsive tissues.^39,83^

Visceral adipose tissue contains large populations of immune cells and their interactions with adipocytes and other cells have a strong impact on adipose tissue function. Within visceral adipose tissue, macrophage numbers increase as the body gains weight.^84^ These macrophages on one hand promote adipose tissue inflammation that has a strong impact on systemic metabolism, but on the other hand they are important for maintaining lipid metabolic homeostasis, clearing dead adipocytes and generating new adipocytes (adipogenesis).^84,85^ Macrophages are thus an essential component of normal adipose tissue function. Within healthy adipose tissue in lean animals, macrophages mainly exhibit the M2 phenotype.^86^ Obesity greatly increases numbers of M1 macrophages in visceral adipose tissue. M1 macrophages impair adipose tissue function in part by promoting insulin resistance and lipolysis. In addition, other subpopulations and/or activation states have been proposed for some adipose-resident macrophages, as their surface phenotypes and gene expression profiles do not fit the typical M1/M2 types.^87–89^

Aging alters the balance of visceral adipose tissue macrophages toward the proinflammatory M1 phenotype in mice.^90^ Aging also increases the numbers of both CD4^+^ and CD8^+^ T cells in visceral fat. Proinflammatory cytokine levels are higher in aged visceral fat, and adipose tissue macrophages from aged mice express higher levels of proinflammatory cytokines.^90,91^ These changes in macrophages may promote adipose tissue inflammation, which may in turn have systemic impact on basal activation of inflammation in the elderly.

The metabolic function of adipose tissue changes with increasing age. Aged adipose tissue becomes less sensitive to insulin, lipolytic stimulation and fatty acids.^92,93^ Moreover, differentiation of preadipocytes is impaired, and the capacity of subcutaneous adipose tissue to store lipids is decreased. Consequently, fat is redistributed to visceral depots and extra-adipose sites, including bone marrow, muscle and liver.^93,94^ Because visceral fat is more prone to inflammation than subcutaneous fat, and accumulation of fat in ectopic tissues (e.g., liver steatosis) promotes inflammation in the affected tissues,^95^ the redistribution of fat may contribute to age-related activation of inflammation. Evidence suggests adipose tissue macrophages promote the generation of new adipocytes (adipogenesis).^96^ It will be important to assess whether the functional alteration of macrophages with age contributes to the diminished adipogenesis seen in the elderly.

## Macrophage origin, microenvironment and aging

For years, it was believed that all tissue-resident macrophages originate from circulating adult blood monocytes, despite evidence that some tissue-resident macrophages are independent of circulating monocytes.^97–99^ However, a recent series of studies demonstrated that many tissue-resident macrophages are established during embryonic development and, in the healthy steady state, persist into the adulthood independently of blood monocytes.^100–105^ Nonetheless, aging may promote replacement of embryo-derived tissue macrophages with monocyte-derived ones.^106^ And while monocyte-derived macrophages are highly plastic and responsive to environmental changes, this shift in the origin of tissue-resident macrophages may still affect the function of macrophages residing within aged tissues. This will need to be directly addressed in future.

The epigenomes and transcriptomes of macrophages are highly plastic, and the tissue microenvironment within certain tissues can transform the enhancer landscape of exogenous macrophages into one mimicking that of resident macrophages.^107,108^ In particular, bone marrow monocytes and fetal macrophages acquire an epigenome and functionality that are very similar to those of the native resident macrophages in colonized tissues.^107–109^ This plasticity suggests age-related changes in the microenvironment alter macrophage function within tissues.^75^ What is more, nearly all microRNAs involved in immune regulation are functionally modulated during aging.^110^ Clearly, a greater understanding of the changes in macrophage origin and the epigenetic changes that occur with aging is needed. For instance, macrophages in the elderly exhibit age-related reductions in phagocytosis and antigen-presenting capacity.^29^ It will be important to determine whether changes endogenous to macrophages induce this dysfunction. A population of senescent-associated CD4^+^ T cells exhibits features of cellular senescence and a unique transcriptomic signature partly driven by C/EBPα.^111,112^ It is possible that the age-related functional alterations in macrophages are driven by similar cell-autonomous mechanisms. It will also be important to determine whether aging induces epigenetic changes in monocyte/macrophage progenitors and/or tissue macrophages that maintain macrophage populations through self-renewal, and to test whether such epigenetic alterations modulate macrophage function. Because macrophages are so susceptible to environmental cues, it will be important to examine the interplay between cell autonomous and externally driven mechanisms to elucidate age-associated changes in macrophages. In future studies, it will also be important to assess the causative link between age-related alterations in the function of macrophages and the onset and progression of chronic inflammatory diseases.

## Perspectives

As we have summarized here, age-related changes in immunity are characterized by both impairment of adaptive immunity and activation of low-grade chronic inflammation. The paradoxical activation of chronic inflammatory states very likely contributes to the progression of age-associated diseases. Macrophages not only promote inflammation and tissue dysfunction but also are essential for resolution and healing of inflammation, as well as maintenance of tissue homeostasis. Accordingly, macrophages appear to contribute crucially to the paradoxical activation of basal chronic inflammatory states in the elderly and to the progression of age-associated diseases.

Many factors are likely involved in the activation of chronic inflammation in the elderly, as we have seen. These include (1) intrinsic dysregulation of immune cell responses to inflammatory stimuli—i.e., macrophages may be activated at baseline and overreact to certain stimuli. (2) Age-related alterations to the microenvironment. For instance, macrophages may be activated in response to the accumulation of DAMPs brought about by insufficient clearance of dead cells. In some cases, this may reflect the macrophages’ diminished phagocytotic capacity. However, increased cell death induced by cellular senescence and stress (e.g., metabolic stress: obesity and hyperlipidemia) may also overwhelm the capacity of the phagocytotic system.^21^ (3) Systemic factors, such as metabolic and hormonal signals and inflammation in distant tissues (e.g., adipose tissue). Proinflammatory cytokines and free fatty acids secreted from inflamed adipose tissue can activate inflammation in distant tissues.^113,114^ Age-related changes in adiposity and hormones (e.g., estrogen) may also have a systemic impact on inflammation. (4) Changes in macrophage subtype and origin. Macrophages can assume diverse phenotypes and their epigenomes are highly plastic. Aging may shift the balance among macrophages. Moreover, age may promote replacement of embryo-derived tissue macrophages with monocyte-derived ones. Many other factors, including nutrients, gut microbiota and tissue remodeling are also likely involved.^4^ For instance, improper tissue remodeling (e.g., extensive fibrosis) may impair tissue function and result in the generation of endogenous proinflammatory stimuli such as extracellular matrix molecules and their degradation products (matrikines).^4^ Macrophages are crucially involved in all these processes, which impair the proper resolution of inflammation. As such, a better understanding of the changes in macrophage function, phenotype and epigenome that occur with aging will not only shed a new light on inflammaging, but also lead to identification of new therapeutic targets for the treatment of age-associated ailments.

## Figures and Tables

**Table 1:** Role of macrophage in metabolic control

*Metabolic tissue*	*Resident macrophage*	*Physiological/pathological role*
Hypothalamus	Microglia	Appetite control
Liver	Kupffer cell	Control of hepatocyte metabolism
		Liver steatosis and fibrosis
		Insulin resistance
Pancreatic islet	Resident macrophage	β cell development
		Islet inflammation and β cell dysfunction
Adipose tissue	Resident macrophage	Lipid handling
		Adipogenesis
		Insulin resistance
Skeletal muscle	Resident macrophage	Regeneration
		Insulin resistance

Resident macrophages in metabolic organs are crucial to metabolic control in the steady state. Impairment in the physiological functions of tissue-resident macrophages, which can occur with aging, may contribute to age-related organ dysfunction. Macrophages may also mediate chronic inflammatory processes and tissue dysfunction in obesity.
